# A Rationale for Drug Design Provided by Co-Crystal Structure of IC261 in Complex with Tubulin

**DOI:** 10.3390/molecules26040946

**Published:** 2021-02-10

**Authors:** Jinghong Xian, Faqian Bu, Yuxi Wang, Fangyi Long, Zhixiong Zhang, Chengyong Wu, Yiran Tao, Ting Wang, Guan Wang

**Affiliations:** 1 Department of Clinical Research Management, Innovation Center of Nursing Research, Nursing Key Laboratory of Sichuan Province, West China Hospital, Collaborative Innovation Center of Biotherapy, Sichuan University, Chengdu 610041, China; xianjinghong@wchscu.cn (J.X.); 18224412763@163.com (F.B.); allenwu1991@163.com (C.W.); 2 Precision Medicine Research Center, Department of Respiratory and Critical Care Medicine, West China Hospital, Sichuan University, Chengdu 610041, China; yuxiwang@scu.edu.cn (Y.W.); zxzhang1022@163.com (Z.Z.); taoyr@163.com (Y.T.); 3 Department of Pharmacy, Sichuan Cancer Hospital & Institution, Sichuan Cancer Center, School of Medicine, University of Electronic Science and Technology of China, Chengdu 610041, China; longfangyi1986@sina.com

**Keywords:** IC261, tubulin, colchicine binding site, crystal structure, drug design

## Abstract

Microtubules composed of α/β tubulin heterodimers are an essential part of the cytoskeleton of eukaryotic cells and are widely regarded as targets for cancer chemotherapy. IC261, which is discovered as an ATP-competitive inhibitor of serine/threonine-specific casein kinase 1 (CK1), has shown its inhibitory activity on microtubule polymerization in recent studies. However, the structural information of the interaction between tubulin and IC261 is still unclear. Here, we provided a high-resolution (2.85 Å) crystal structure of tubulin and IC261 complex, revealed the intermolecular interaction between tubulin and IC261, and analyzed the structure–activity relationship (SAR). Subsequently, the structure of tubulin-IC261 complex was compared with tubulin-colchicine complex to further elucidate the novelty of IC261. Furthermore, eight optimal candidate compounds of new IC261-based microtubule inhibitors were obtained through molecular docking studies. In conclusion, the co-crystal structure of tubulin-IC261 complex paves a way for the design and development of microtubule inhibitor drugs.

## 1. Introduction

Microtubules are intrinsically dynamic polymers, containing two highly homologous proteins—α- and β-tubulin heterodimers, which assemble into protofilaments in a head-to-tail form [1,2]. As a core component of the eukaryotic cytoskeleton, microtubules are involved in many physiological activities in cells, for instance, the maintenance of cell shape and motility, formation of the spindle during cell mitosis, intracellular material transport during interphase, signal transduction, and other essential processes [3,4]. Being involved in such complex cell activities, microtubules are widely regarded as attractive targets for anticancer chemotherapy, and a large number of small molecule inhibitors that interfere with microtubule dynamics have been discovered. They inhibit assembly or inhibit disassembly, showing extraordinary anticancer activity [5]. According to the different binding sites of microtubules, these drugs can be mainly classified into six families: colchicine binding site inhibitors (CBSIs), vinca site inhibitors, maytansine site inhibitors, pironetin site inhibitors, laulimalide/peloruside site inhibitors, and taxane site inhibitors [6,7,8,9]. Among them, the first four groups are “destabilizing” drugs that inhibit assembly, while the last two groups are the “stabilizing” drugs that inhibit disassembly [5]. Although these inhibitors have significant effects, the emergence of multi-drug resistance limited their application and research, especially paclitaxel microtubule inhibitors [10]. Compared with vinblastine and paclitaxel microtubule inhibitors, due to various reasons (such as cytotoxicity), CBSIs have not been commercialized for anticancer treatment, although they have been extensively investigated in laboratories as well as in clinical trials over the past decade [11]. Therefore, exploring novel microtubule-targeting agents is urgent.

**Figure 1:**
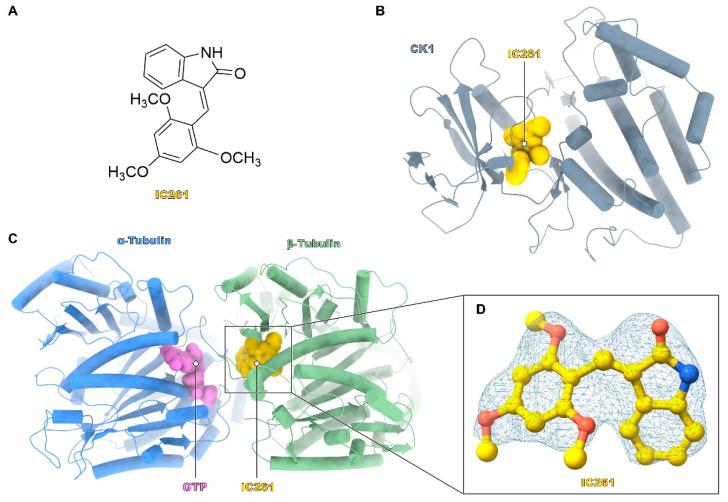
Crystal structure of IC261 complexed with casein kinase 1 (CK1) and tubulin. (**A**) Chemical formula of IC261. (**B**) Binding mode of the CK1-IC261 complex structure. The CK1 is shown in grey and IC261 in yellow. (**C**) The overall structure of tubulin-IC261 complex. The α-tubulin is shown in blue, β-tubulin in green, guanosine triphosphate (GTP) in purple, and IC261 in yellow. (**D**) The electron densities of IC261. The Fo-Fc map is shown grey and contoured at 2.0 σ.

The serine/threonine-specific casein kinase 1 (CK1) family is widely expressed in cells, playing a key role in the regulation of many physiological processes, including Hedgehog and Wnt signaling pathways [10], PERIDO 2 (PER2) protein [12], cell apoptosis and necroptosis [13,14], etc. The 3-((2,4,6-trimethoxyphenyl) methylidenyl)-indolin-2-one (IC261) turned out to be a specific CK1 inhibitor (Figure 1A), which competes with ATP and selectively binds with CK1. The co-crystallization structure of CK1-IC261 (Protein Data Bank Identity (PDB ID): 1EH4) was reported by Mashhoon et al. in 2000 (Figure 1B) [15]. Moreover, in previous studies, it has been shown that IC261 induces microtubule depolymerization by competing with colchicine for binding to tubulin, and its effect may be not mediated by CK1 blockage [16,17], which indicates IC261 may be a microtubule inhibitor.

Encouragingly, BNC105, a selective tubulin polymerization inhibitor targeting the colchicine binding site, is currently undergoing phase II clinical trials [18,19]. It is worth noting that trimethoxyphenyl and benzofuran groups have been demonstrated to be the key functional groups for BNC105 to bind tubulin [19]. Similar to BNC105, IC261 also possesses trimethoxyphenyl group, and shows inhibitory activity against microtubules. As for the indolyl group in IC261, the study of structure–activity relationship (SAR) between derivatives with indole nucleus and tubule interaction explained why the indole structure is indispensable for CBSIs, which also provided proof for IC261 as a CBSI [20]. Nonetheless, the structure information of the interaction between IC261 and microtubules is still unclear, which hinders the design and development of microtubule inhibitors based on IC261, and emphasizes the urgency and necessity of investigating the interaction between tubulin and IC261.

In this study, we identified the crystal structure of IC261 in complex with tubulin at a resolution of 2.85 Å and carried out the structure–activity relationship (SAR) study. Following, eight optimal candidate compounds of microtubule inhibitor drugs were obtained based on the mother nucleus of IC261. Attractively, we found that IC261 and colchicine occupied the same site, and blocked the change in the conformation of the T7 loop. This is different from other colchicine site inhibitors, indicating that IC261 is a new type of microtubule inhibitor.

## 2. Results

### 2.1. Visualizing Binding Modes of Tubulin-IC261 Complex

To obtain the precise description of how IC261 interacts with tubulin, we solved the structure of tubulin in complex with IC261 by X-ray crystallography. As shown in Figure 1A, the structure of IC261 consists of trimethoxyphenyl and oxindole, connected by methylene. Here, we determined the tubulin-bound IC261 structure at 2.85 Å resolution (Figure 1C) using crystals of a protein complex encompassing α/β-tubulin, the stathmin-like protein RB3 and tubulin tyrosine ligase, generally called the T2R-TTL complex. Details of the crystallographic data parameters and refinement statistics are summarized and displayed in Table 1. Then, the position and orientation of the inhibitor IC261 were analyzed, according to the highly unambiguous difference of electronic density map (Figure 1D).

**Figure 2:**
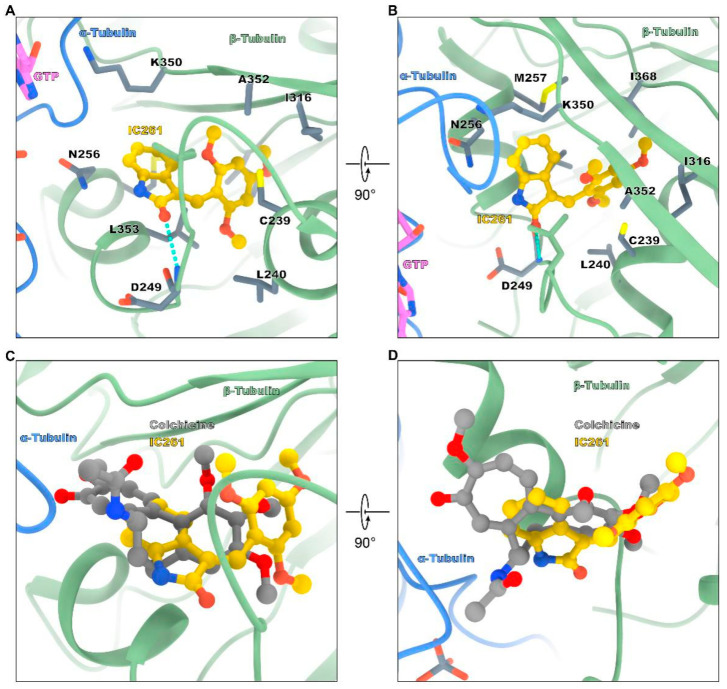
Interactions between IC261 and tubulin, and comparisons of the binding modes between IC261 and colchicine. The two colchicine binding site inhibitors (CBSIs) are shown as sticks. Tubulin, IC261, and GTP are colored as in Figure 1 represented by sticks. Interacting residues of tubulin are shown in stick representation and are labeled. Colchicine is colored in grey. Oxygen atoms of the compounds are colored in red and nitrogen atoms in blue. (**A**,**B**) Intermolecular interactions of the binding mode between IC261 and tubulin. (**C**,**D**) The complex structures of tubulin-IC261 and tubulin-colchicine (PDB ID: 4O2B) are superimposed.

In the crystal complex of tubulin-IC261, the compound occupies a pocket formed by residues of strands S8, S9 and S10, loop T7 and helixes H7 and H8 of β-tubulin and loop T5 of α-tubulin (Figure 2A,B), which is identified as the colchicine binding site [21,22]. IC261 is in hydrophobic contact with the hydrophobic amino acids of Val236, Leu246, Leu253, Ala248, Ala314, and Ala315 of β-tubulin. In detail, the carbonyl group on the pentagonal heterocyclic ring of IC261 forms a conventional hydrogen bond with the side chain of βAsp249. Three methoxy groups on the benzene ring interact with βVal236, βAla314, βAla315 by modest hydrogen bond formed by the carbon and the hydrogen atoms (carbon hydrogen bond), respectively. The pentagonal ring interacts with βLeu246 by pi-sigma interaction. In addition, IC261 interacts with βAla248, βLys252, βLeu253, βLys350 by hydrophobic interaction (pi-amide and pi-alkyl interactions) (Figure 2A,B).

### 2.2. Comparison of the Binding Modes between IC261 and Colchicine

**Figure 3:**
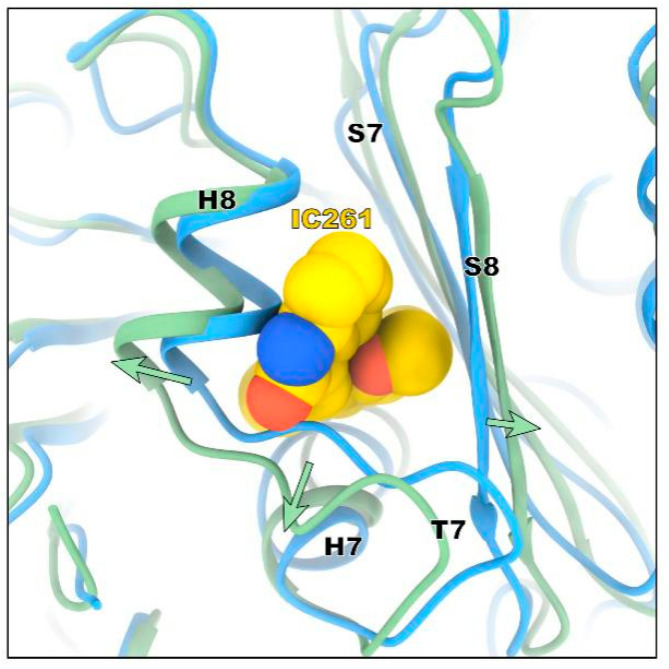
Conformational changes of the colchicine domain when tubulin binds to IC261. IC261 is shown in yellow sphericity. The position and shape of tubulin before and after binding to IC261 are represented by blue and green respectively.

Colchicine, a tricyclic alkaloid extracted from the herbaceous plant *Colchicum autumnale*, is the first compound identified as a microtubule-targeting agent binding to the colchicine site [21]. To compare the structure of tubulin-IC261 and tubulin-colchicine complex, we superimposed the colchicine binding sites of the tubulin-IC261 structure onto the tubulin-colchicine structure (PDB: 4O2B). As seen in the crystal structure, upon binding to the colchicine site, IC261 could roughly overlap on the position of colchicine and occupy the analogous position similar to colchicine (Figure 2C,D). Interestingly, compared with the structure of protofilament tubulin, IC261 induced some significant conformational changes after binding (Figure 3), which was similar to the previously reported colchicine site microtubule inhibitors [23]. These results indicated that IC261 may be a new colchicine site microtubule inhibitor.

In the assembled microtubules, tubulin exists in the form of a straight configuration, and curve tubulin dimers are basic characteristics in the depolymerized state. The most typical feature upon ligand binding to the colchicine site is the conformational change of the T7 loop, a switch that is needed to free up space for ligand binding. Colchicine inhibits the assembly of microtubule via binding to the β-tubulin, preventing it to adopt a straight structure from curve one [21,22]. Similarly, the binding of IC261 inhibits the assembly of microtubules in the same way, hindering transition of tubulin from curve to straight, which is a necessary process for the formation of microtubules. The “curve-to-straight” conformational transition is characterized by strands S8, S9 and others, which move closer to helix H8 [24]. Upon binding with IC261, the strand S8, the helix H8, and the T7 loop move away from each other, preventing the T7 loop going from the curve state to the straight-line state (Figure 3). Taken together, the binding mode of IC261 and tubulin is similar to that of colchicine, which prevents the conformational change the T7 loop, thereby inhibiting the assembly of microtubules. 

### 2.3. Insights into Drug Design and Molecular Docking Studies

**Figure 4:**
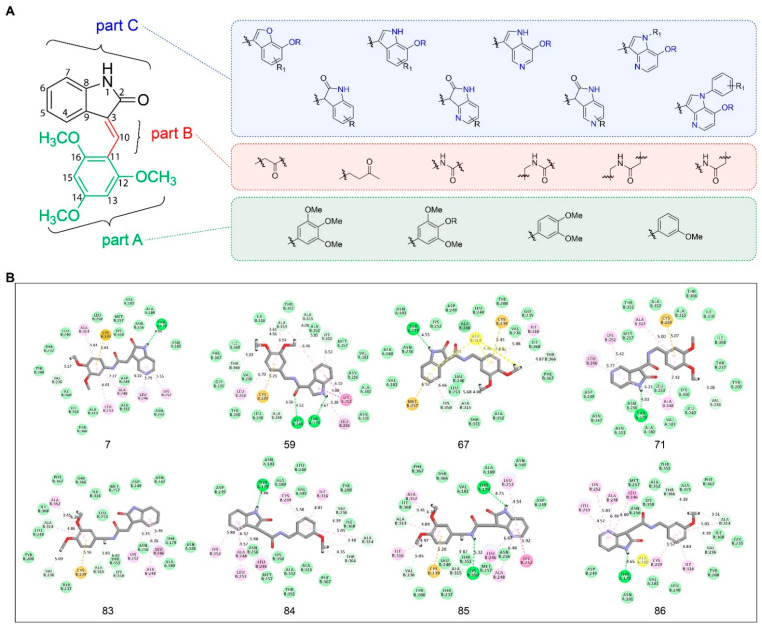
Insights into drug design and molecular docking studies. (**A**) IC261 molecular Design Strategy. (**B**) Three-dimensional (3D) schematic diagram of the binding model of eight candidate compounds with tubulin. The eight candidate compounds are shown as grey sticks, the oxygen atoms are shown in red, and the nitrogen atoms are shown in blue. Interactions between the molecules and amino acid residues are labeled with dotted lines, van der Waals is shown in green, carbon hydrogen bond is shown in light green, conventional hydrogen bond is shown in bottle green, pi-alkyl is shown in pink, amide-pi stacked is shown in deep pink, and pi-sulfur is shown in yellow.

The structure and interaction between tubulin and IC261 described here may provide insights to the design and development of drugs based on IC261. In order to obtain more effective microtubule inhibitors, we divided the molecule IC261 into Part A, Part B, and Part C (Figure 4A) based on the interactions and the binding model between IC261 and tubulin. The methoxy group of 2,4,6-trimethoxyphenyl ring (Part A) has a similar structure to the 2,3,4-trimethoxyphenyl group of colchicine [25], which forms carbon hydrogen bond interactions with tubulin, suggesting that it is tolerable to adjust the position of the substituent of the methoxy group, but is not suitable for larger modifications. The C-C bond in the connection bridge (Part B) does not interact with any key amino acids, which shows the rationality of introducing amide, carbonyl, or other groups to explore the SAR and extend the chain appropriately. Considering the volume of microtubule cavity, it is not recommended to make the chain too long to occupy the space. Herein, we suggest retaining the penta-heterocycle of oxindole (Part C) as it forms a hydrogen bond with the amino acid residues βAsp249. Nevertheless, it is feasible to introduce halogen, amino, hydroxyl or other suitable groups on the benzene ring, to replace it with heterocycles, such as pyridine, or to amplify it into a heptatomic ring to increase anticancer activity. Based on the above analysis, 97 candidate compounds, including IC261, were designed (Figure 4A and Table S1).

Before the molecular docking studies of these 97 candidate compounds, we performed a redocking analysis on IC261 for the purpose of proving the credibility of our method. The detailed redocking method and results are seen in Supplementary Materials (Figure S1 and S2). According to the scoring and docking mode analysis, eight compounds showed stronger interactions with tubulin than IC261 (CDOCKER Interaction Energy: IC261-85<IC261-67<IC261-7<IC261-83<IC261-59<IC261-71<IC261-86<IC261-84<IC261). The complex systems formed by eight candidates and tubulin were more stable than that of IC261 (CDOCKER Energy: IC261-59<IC261-83<IC261-86<IC261-84<IC261-85<IC261-67<IC261-71<IC261-7<IC261) (Table 2). By analyzing the interactions of the eight molecules and tubulin, we can figure out that the introduction of the amide bond in Part B promotes the flexibility of IC261, which makes the amino group on the five-member heterocyclic ring (Part C) form a stable hydrogen bond with αThr179. This hydrogen bond is important for the activity of colchicine site microtubule inhibitors and is consistent with the interaction between the colchicine site microtubule inhibitors and other previously reported microtubule inhibitors. The location and number of methoxy group in part A have no significant effect on the activity of IC261. The Part C of eight compounds superior to IC261 was almost the same as that of IC261, which indicates that Part C plays a pivotal role for the activity of IC261 (Figure 4B). Summarily, the docking studies have shown that Part A and Part C are necessary to bind to tubulin, indicating that these two parts should not be significantly modified, while Part B recommends the introduction of the amide bonds. In conclusion, the structure and interaction analysis between tubulin and IC261 and the results of molecular docking studies may contribute to further studies on inhibition activities against microtubules of IC261 and its derivatives.

## 3. Materials and Methods

### 3.1. Special Reagents

Porcine brain tubulin (Catalog#T-238P) was gained from Cytoskeleton. IC261 (Catalog HY-12774) was purchased from MedChem Express.

### 3.2. Protein Expression and Purification

The T2R-TTL complex, including α- and β-tubulin, one stathmin-like domain of RB3 (RB3–SLD) and one tubulin tyrosine ligase (TTL), was produced as the previously reported method [26,27]. Porcine brain tubulin (Cytoskeleton, Catalog # T-238P) was diluted to 10 mg/mL in the buffer (80 mM Pipes, 2.0 mM MgCl_2_, 0.5 mM EDTA and 1 mM GTP, pH 6.9) and stored at −80 °C until use. The RB3–SLD protein was overexpressed in Escherichia coli strain BL21 (DE3), and purified primarily by anion-exchange chromatography (QFF; GE Healthcare, Chicago, IL, USA) and then by gel filtration (Superdex 75; GE Healthcare, Chicago, IL, USA). The peak fractions of the purified RB3–SLD protein were concentrated to 10 mg/mL and stored at −80 °C until use. TTL was overexpressed in Escherichia coli strain BL21(DE3) in the same way, and purified by nickel-affinity chromatography followed by gel filtration (Superdex 200; GE-Healthcare, Chicago, IL, USA). The purified TTL in the solution (Bis-Tris propane, 200 mM NaCl, 2.5 mM MgCl_2_, 5 mM β-mercaptoethanol, and 1% glycerol, pH 6.5) was concentrated to 20 mg/mL and stored at −80 °C until use. The purity of RB3 and TTL was analyzed by sodium dodecyl sulfate−polyacrylamide gel electrophoresis and stained with Coomassie Brilliant Blue. The T2R-TTL complex was produced by mixing tubulin, RB3–SLD and TTL in the solution (5 mM tyrosine, 10 mM DTT, and 1 mM β, γ-methyleneadenosine 50-triphosphate disodium salt) at a tubulin/RB3-SLD/TTL mass ratio of 1:1.2:1.3. The complex was concentrated to 20 mg/mL at 4 °C.

### 3.3. Crystallization and Crystals Soaking

The crystals of T2R-TTL complex were obtained by hanging drop vapor diffusion method at 20 °C, by mixing the complex with the solution containing 6% PEG4000 (poly ethylene glycol 4000), 8% glycerol, 0.1 M MES (pH 6.7), 30 mM CaCl_2_, and 30 mM MgCl_2_. Seeding method was used to gain single crystals. The crystal was appearing when incubating for 3 days, and coming up the maximum dimensions within 5 days. For crystal soaking, 0.1 μL of IC261 (10 mM in DMSO) was added to a 2 μL crystal solution for 5 h at 20 °C before data collection. 

### 3.4. Data Collection and Structure Determination

The crystals were soaked in the cryoprotectant, which was supplemented with 20% (*v/v*) glycerol for a few seconds, then mounted in nylon loops and flash-cooled in liquid nitrogen. Diffraction data were collected at beamline BL18U1 from Shanghai Synchrotron Radiation Facility (SSRF) (Shanghai, China). Data were indexed, integrated, and scaled by HKL2000 program package [28]. The structures of T2R-TTL-ligand were built by molecular replacement program PHASER regarding the T2R-TTL structure (PDB ID: 4I55) as template. The manual model was built with COOT [29]. Refinement was performed by PHENIX [30]. The model quality was checked on MolProbity [31]. All the figures were made by PyMOL^TM^ (Version 1.8 Schrödinger, LLC, New York, NY, USA, 2017). 

### 3.5. Molecular Docking

CDOCKER and MM/GBSA module of Discovery Studio 4.5 was used for molecular docking study. CDOCKER is a molecular docking method based on CHARMM36 force field, which can produce high precision docking results. The receptor is held rigid while the ligands are allowed to flex during the docking process. For each complex pose, the CHARMM energy (interaction energy plus ligand strain) and the interaction energy, which indicated ligand-binding affinity, were calculated. The crystal water molecules were generally removed in rigid and semi-flexible docking process, since the fixed water molecules might affect the formation of receptor-ligand complex. Next, the water molecules were removed and hydrogen atoms were added to the protein. In order to prove the reliability of the combination mode, the initial compound IC261 was extracted from the binding site and then re-docked into the crystal structure of microtubule. The CHARMM36 force field was used for both receptors and ligands. The binding site sphere of microtubule was defined as the region that came within radius 7 Å from the geometric centroid of the ligand IC261. During the docking process, the ligands were allowed to bind to the residues within the binding site spheres. The structures of IC261 and IC261 derivates were prepared and docked into the binding pocket of microtubule. The CDOCKER process was performed. Ten docking poses were generated for each ligand and the best pose was selected based on high docking scores and appropriate docking orientations. Different poses of each test molecules were generated and analyzed on the basis of CDOCKER interaction energy, MM/GBSA free energy, respectively.

## 4. Discussion

CBSIs have been extensively investigated over the past decade in laboratory and clinical trials [10]. In this study, we found IC261 as a novel CBSI using X-ray crystal diffraction. According to the SciFinder, only the modification of IC261 to inhibit CK1 has been studied [32], but there is no pharmacochemical study on IC261 inhibiting tubulin to show anticancer effect, which indicates that it is meaningful to conduct a pharmacochemical study on this compound.

The complex of tubulin-IC261 has been obtained by crystals soaking, the co-crystal structure data has been collected, and the structure has been determined at 2.85 Å resolution. In our research, we found that IC261 can occupy the colchicine-binding site of tubulin, and the interaction between the trimethoxyphenyl group of IC261 and tubulin, as well as the hydrogen bond formed by carbonyl group and the side chain of βAsp249, is important to its inhibitory activity. The structural comparison between the tubulin-IC261 and tubulin-colchicine complex showed that IC261 roughly overlaps on the position of colchicine and occupies the analogous position similar to that of colchicine, indicating analogous binding pattern of IC261 and colchicine. Indeed, upon binding with IC261, the strand S8, the helix H8 and the T7 loop move away from each other, preventing the T7 loop going from the curve state to the straight-line state; thus, inhibiting microtubule assembly. The interaction of tubulin and IC261 provides a direction of drug design. The methoxy groups of the 2,4,6-trimethoxyphenyl ring (Part A) in IC261 is important for the inhibitory activity and no more modification is suggested, except the adjustment of the methoxy substituent position. The connection bridge (Part B) does not interact with any key amino acid, which explains the rationality of the modification. While the penta-heterocycle of oxindole (Part C) forms hydrogen bond with tubulin, and we recommend keeping it. Guiding by the rationale, we have designed 97 candidate compounds, and upon molecule docking studies, eight of them have greater interactions with tubulin than IC261. The docking studies revealed that Part A and Part C are necessary for tubulin binding, which indicates that these two parts should not be greatly modified, while the introduction of the amide bond in Part B was suggested. In follow-up research, we will design and synthesize more small-molecule drug candidates (including the above mentioned eight compounds) with high targeting ability, high activity, and novel binding mode based on the obtained SAR, and then conduct research on pharmacodynamics, pharmacology, and structural biology evaluation. 

## 5. Conclusions

Here we report a 2.85 Å crystal structure of tubulin complexed with IC261, which is the first crystal structure of tubulin-IC261 complex. In the crystal structure, IC261 makes hydrophobic contact with hydrophobic amino acid of Val236, Leu246, Leu253, Ala248, Ala314, and Ala315 of β-tubulin. In detail, the carbonyl group on the pentagonal heterocyclic ring of IC261 forms a strong hydrogen bond with βAsp249. Three methoxy groups on the benzene ring interact with βVal236, βAla314, βAla315 by carbon hydrogen bonds, respectively. The pentagonal ring interacts with βLeu246 by pi-sigma interaction. In addition, IC261 interacts with βAla248, βLys252, βLeu253, βLys350 by pi-amide and pi-alkyl interactions. The structure and interaction between tubulin and IC261 disclosed here may benefit the design and development of drugs on the basis of IC261. We divided the molecule IC261 into Part A, Part B, and Part C based on the interactions and the binding mode between IC261 and tubulin. After simulation docking, we found that it could be a good choice to introduce amide bond for IC261 in Part B. The introduction of the amide bond in Part B promoted the flexibility of IC261, which made the amino group form a stable hydrogen bond with αThr179. This hydrogen bond may be important for the activity of colchicine site microtubule inhibitors and was consistent with the previously reported interaction between colchicine site microtubule inhibitors and microtubules. Based on these, we obtained a total of eight candidate compounds superior to IC261. We plan to go further on these candidate compounds through synthetical, pharmacological, and pharmacological studies.

## Figures and Tables

**Table 1:** Data collection and refinement statistics for X-ray structure.

Data	Tubulin/IC261
X-Ray Source	SSRF-BL18U1
Integration Package	HKL2000
**Data Collection**	
Resolution Range (Å)	49.87–2.85
Space Group	P 212121
Unit Cell (Å, °)	105.1 157.7 182.1 90, 90, 90
Total Reflections	931,632
Unique Reflections	71,664
Redundancy	13.3 (13.3)
Completeness (%)	100.0 (100.0)
Mean I/sigma (I)	15.5 (2)
Rmerge	0.177 (0.933)
CC1/2	0.995 (0.833)
**Structure Refinement**	
R-Factor/R-Free	0.1864/0.1903
Root Mean Square (RMS) (Bonds)	0.01
RMS (Angles)	0.17
No. of Atoms	17,757
Protein	17,520
Ligands	237
Waters	0
Average B-Factor	50.23
Protein	50.18
Others	50.83
**Ramachandran Plot Statistics**	
Most Favored Regions (%)	96.84
Allowed Regions (%)	3.16
Disallowed Regions (%)	0

**Table 2:** Top eight ranked compounds with higher CDOCKER scores than IC261.

ID	CDOCKER Energy	CDOCKER Interaction Energy
IC261-85	−28.35	−55.01
IC261-67	−27.68	−53.24
IC261-7	−19.01	−52.47
IC261-83	−29.90	−52.16
IC261-59	−33.53	−51.44
IC261-71	−24.69	−51.38
IC261-86	−29.65	−51.33
IC261-84	−28.82	−51.13
IC261	−5.65	−50.76

## Data Availability

The atomic coordinates and structure factors for tubulin-IC261 complex have been deposited in the Protein Data Bank under accession code 7DB9.

## Supplementary Materials

The following are available online, Figure S1: The superimposition of the original and the redocking conformations of IC261, Figure S2: The root mean square variance (RMSD) between the docking conformation and the original conformation, Table S1: The CDOCKER Results of IC261.

## Sample Availability

Samples of the tubulin-IC261 complex are available from the authors.
