# Supplementary material for: A Rationale for Drug Design Provided by Co-Crystal Structure of IC261 in Complex with Tubulin
Source: Molecules. 2021 Feb 10;26(4):946. doi: 10.3390/molecules26040946 (PMC7916759; doi:10.3390/molecules26040946)
Supplement: Supplementary file 1 [file molecules-26-00946-s001.pdf]

Supplementary Materials:

Table S1. The CDOCKER Results of IC261

Structure	Number	-DOCKER ENERGY	-DOCKER INTERACTION ENERGY
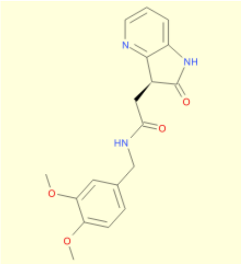	IC261-85	28.3476	55.0063
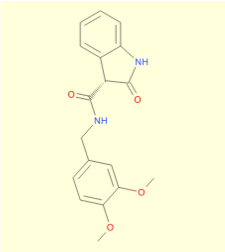	IC261-67	27.677	53.243
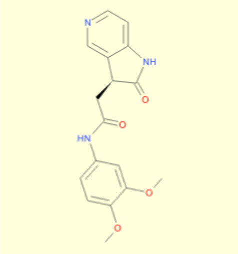	IC261-7	19.0067	52.4687
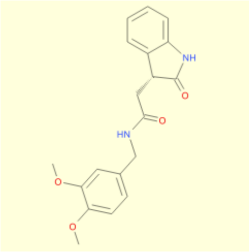	IC261-83	29.8994	52.1593
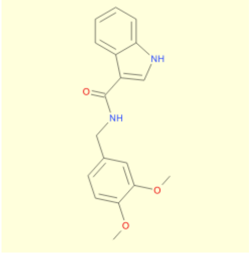	IC261-59	33.5258	51.444

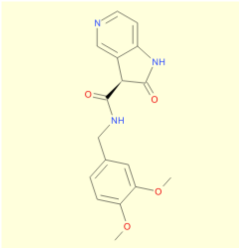	IC261-71	24.6936	51.3824
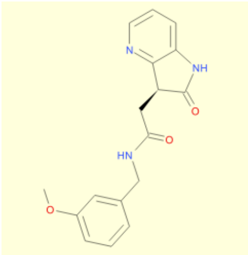	IC261-86	29.6497	51.3155
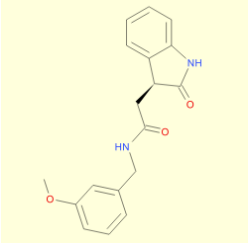	IC261-84	28.8156	51.1278
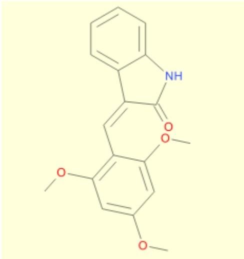	IC261	5.65048	50.7624
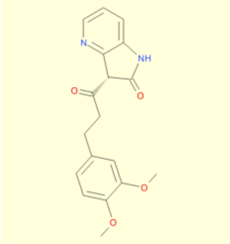	IC261-37	22.5828	50.4947
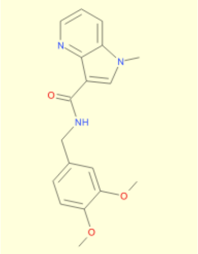	IC261-63	31.6164	50.3925

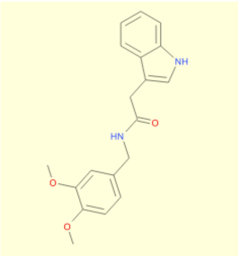	IC261-75	37.0215	50.2725
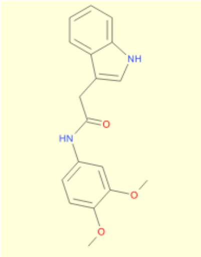	IC261-91	34.0401	50.1522
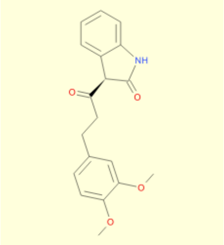	IC261-35	18.2462	50.117
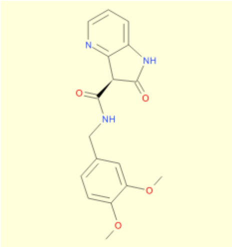	IC261-69	19.3092	50.0616
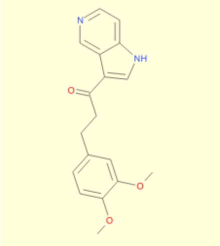	IC261-29	27.6935	50.0145
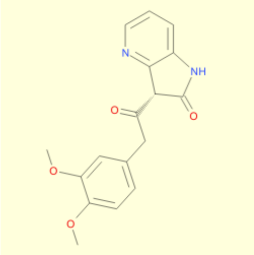	IC261-21	21.1081	49.9331

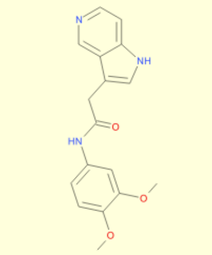	IC261-93	31.6764	49.7824
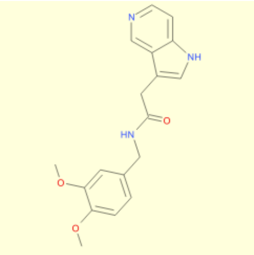	IC261-77	35.0064	49.525
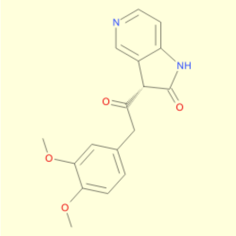	IC261-23	19.7973	49.3668
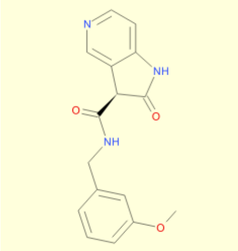	IC261-72	27.4504	49.3175
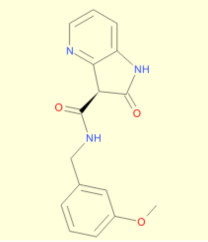	IC261-70	25.6619	49.0729
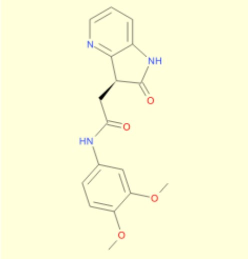	IC261-5	14.9828	48.8917

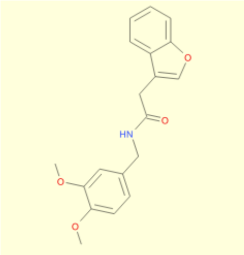	IC261-73	36.5278	48.7393
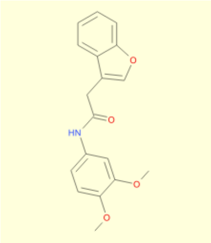	IC261-89	33.3856	48.5196
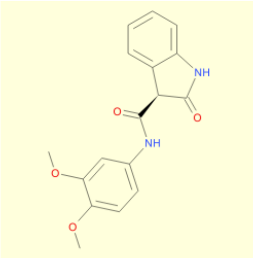	IC261-51	14.6334	48.4993
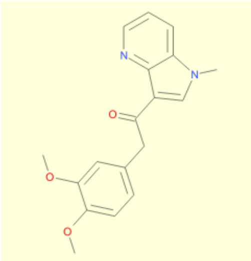	IC261-15	18.4478	48.3111
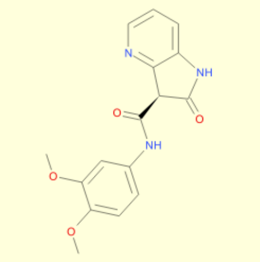	IC261-53	13.2257	48.2912
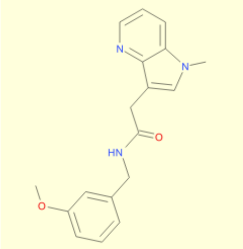	IC261-80	39.5839	48.2642

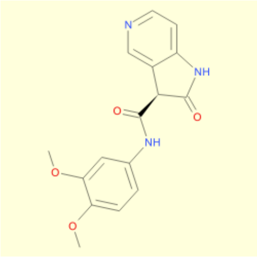	IC261-55	15.9335	47.8623
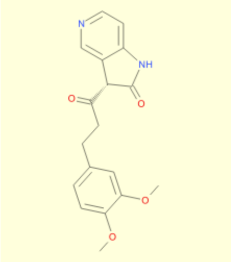	IC261-39	20.5313	47.7242
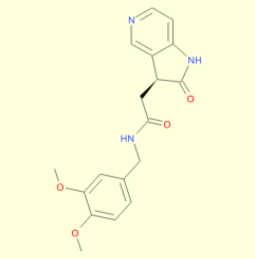	IC261-87	22.1903	47.6339
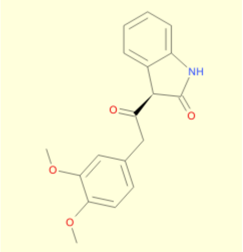	IC261-19	17.0567	47.3126
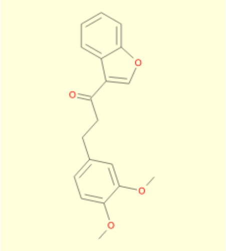	IC261-25	28.9044	46.9493
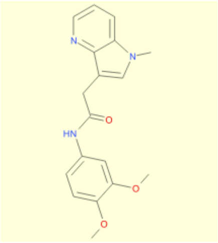	IC261-95	27.1931	46.8107

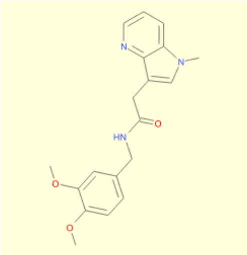	IC261-79	27.7733	46.513
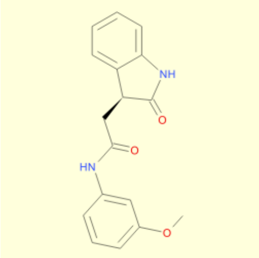	IC261-4	22.2507	46.3597
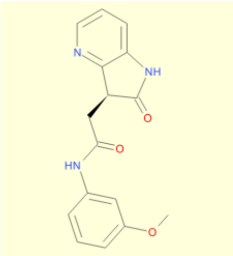	IC261-6	22.3052	46.3295
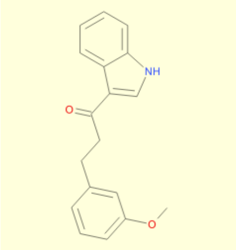	IC261-28	33.5922	46.2708
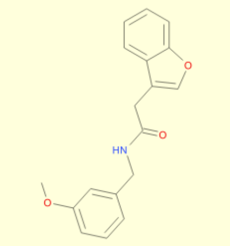	IC261-74	37.3355	46.076
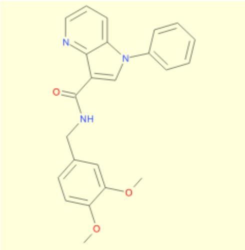	IC261-65	3.35754	46.0378

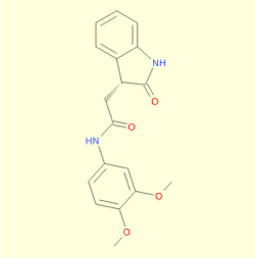	IC261-3	15.7762	45.9616
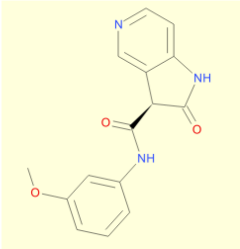	IC261-56	18.4919	45.9057
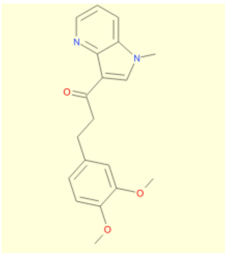	IC261-31	23.2878	45.8359
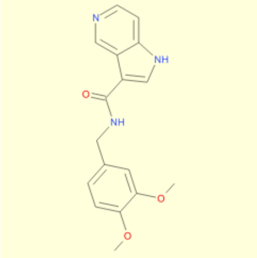	IC261-61	27.9934	45.8225
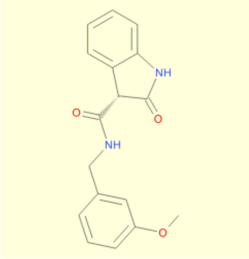	IC261-68	25.3538	45.8084
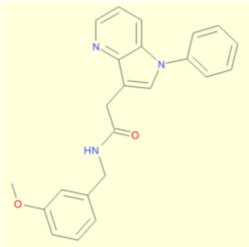	IC261-82	12.8172	45.7401

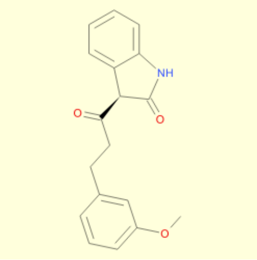	IC261-36	26.2968	45.4971
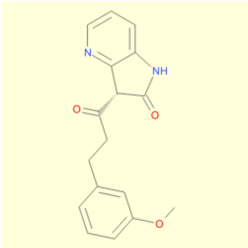	IC261-38	21.5559	45.4151
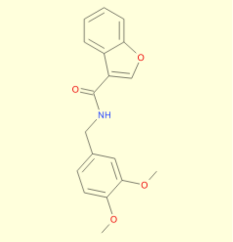	IC261-57	27.9501	44.6775
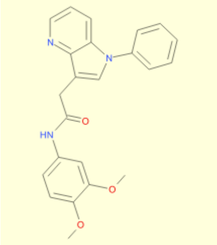	IC261-97	-0.167742	44.5535
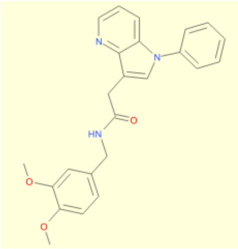	IC261-81	3.78554	44.3331
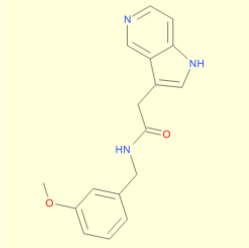	IC261-78	34.9892	44.2238

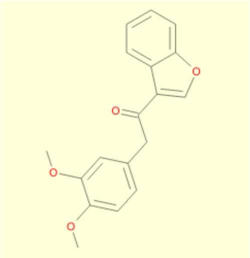	IC261-9	25.1157	44.0386
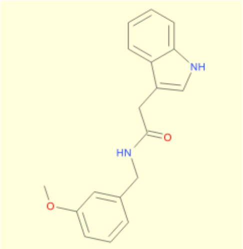	IC261-76	35.5865	43.9872
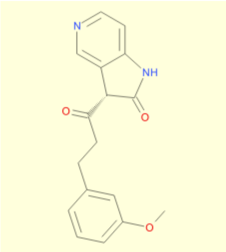	IC261-40	22.5903	43.8156
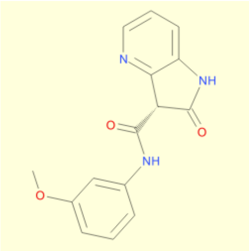	IC261-54	16.6774	43.7243
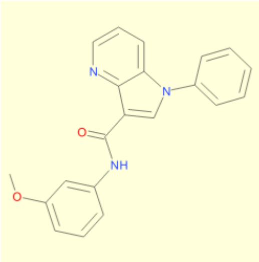	IC261-50	2.76347	43.5349
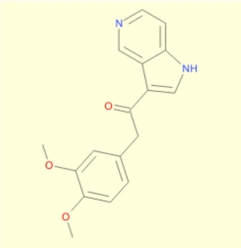	IC261-13	20.0579	43.5217

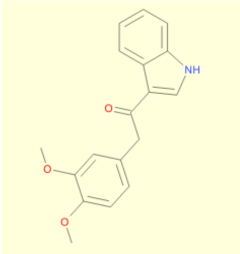	IC261-11	22.9803	43.5167
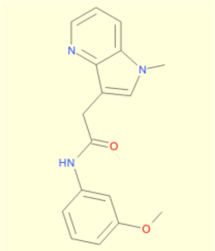	IC261-96	30.1652	43.4767
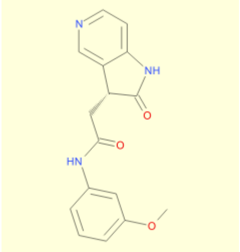	IC261-8	17.2573	43.2851
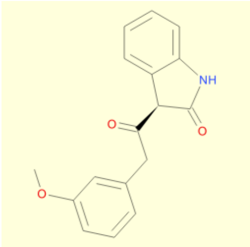	IC261-20	21.4302	42.9968
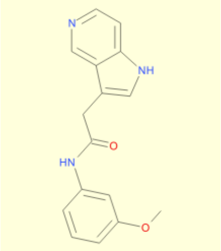	IC261-94	27.4673	42.8824
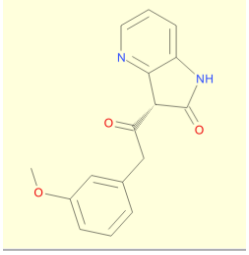	IC261-22	19.4972	42.8673

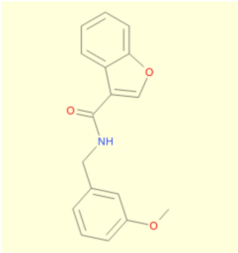	IC261-58	31.4427	42.7167
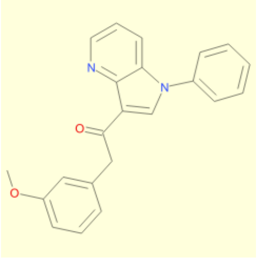	IC261-18	1.02852	42.5954
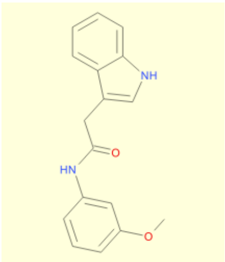	IC261-92	31.0717	42.5683
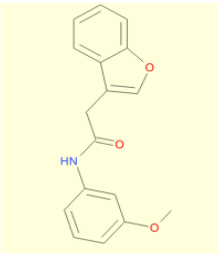	IC261-90	32.0684	42.2944
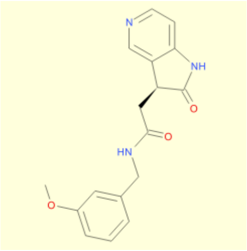	IC261-88	23.9362	41.8312
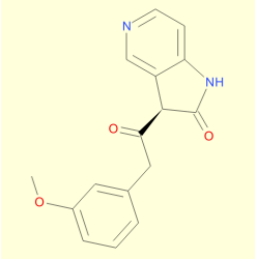	IC261-24	19.2852	41.5083

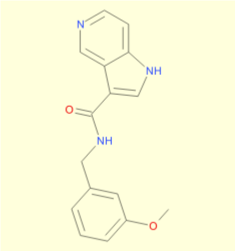	IC261-62	30.4188	41.4802
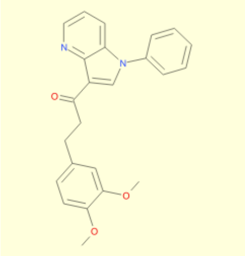	IC261-33	-7.97166	41.4309
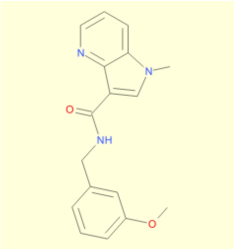	IC261-64	26.9931	41.358
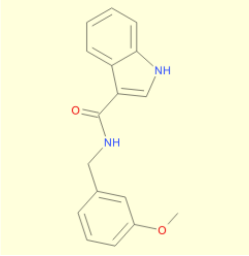	IC261-60	29.783	41.1029
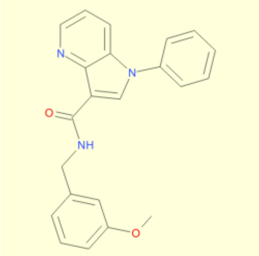	IC261-66	-0.726723	41.0067
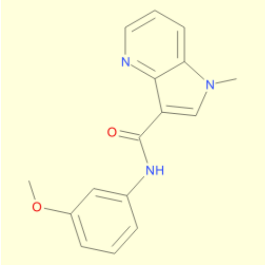	IC261-48	24.3173	40.8294

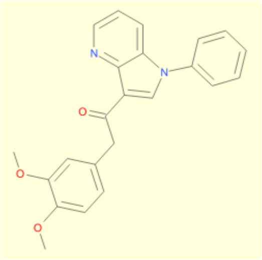	IC261-17	-9.05777	40.5744
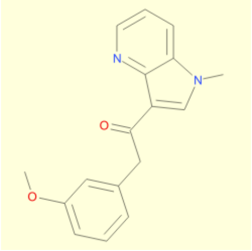	IC261-16	25.3145	40.0144
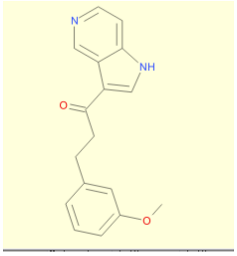	IC261-30	24.8698	39.9366
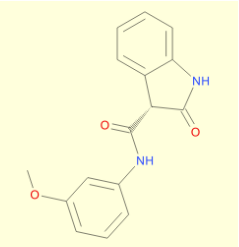	IC261-52	15.4301	39.9018
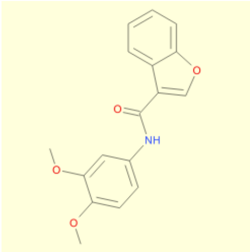	IC261-41	19.0966	39.2201
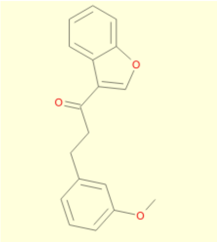	IC261-26	26.5189	39.1905

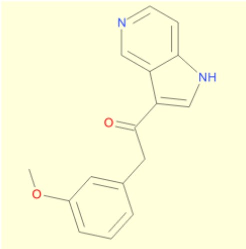	IC261-14	25.4409	39.0956
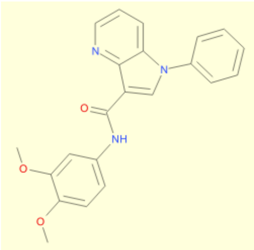	IC261-49	-12.523	38.7287
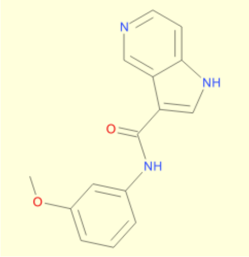	IC261-46	25.1365	38.1879
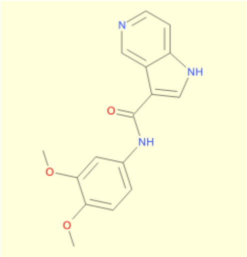	IC261-45	17.655	38.143
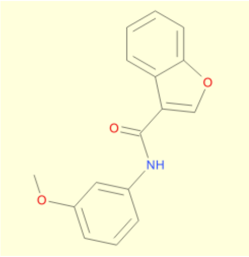	IC261-42	25.9982	37.8148
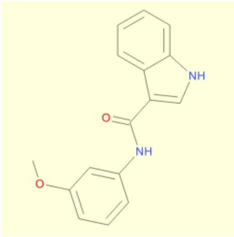	IC261-44	23.4286	37.5918

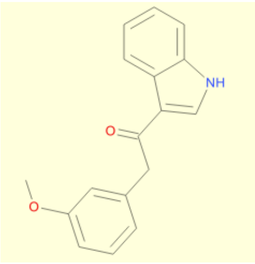	IC261-12	26.4566	37.4763
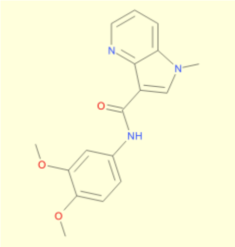	IC261-47	14.8729	37.4291
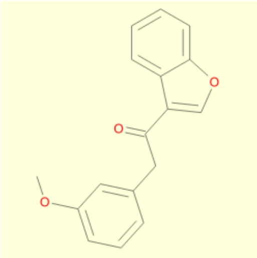	IC261-10	27.1349	37.0368
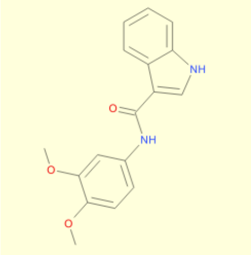	IC261-43	15.9403	37.016
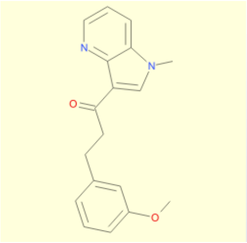	IC261-32	22.8603	36.9303
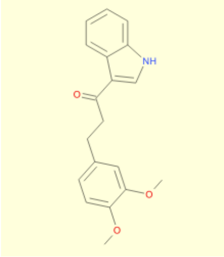	IC261-27	16.4185	36.2056

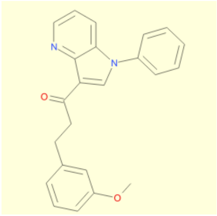	IC261-34	-11.9472	32.5087
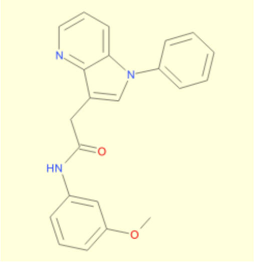	IC261-2	-0.037182	31.95

Establinshment CDOCKER Docking Method

The CDOCKER module of Discovery Studio 2016 was used for docking. Firstly, the crystal of IC261-tubulin complex was imported into the software, the solvent water molecules were removed, and a group of α-tubulin, β-tubulin and the IC261 molecule were retained. Tools-Macromolecules-Prepare Protein was selected to prepare the tubulin structure. Secondly, selected the IC261 molecule and pasted it into the new window named "Original-IC261" as the original crystal conformation. Selected Prepare Ligands to prepare the structure for the IC261 molecule. Thirdly, clicked IC261 and selected Tools-Receptor Ligand Interactions-Define and Edit Binding Site-From Current Selection to define the docking site. The prepared tubulin structure was checked as a receptor and IC261 as a ligand to implement docking in Tools-Receptor Ligand Interactions-Dock Ligand-CDOCKER.

The original crystal structure of the IC261 molecule conformation was copied into 10 conformation lists generated by redocking. Then selected the conformation and clicked Structure-RMSD-Set Reference. Selected all conformations and clicked Structure-RMSD-Heavy Atoms to calculate the root mean square variance (RMSD) between the docking conformations and the

original conformation. The superimposition of the original structure and the redocking conformations are shown in Figure S1 and the RMSD is shown in Figure S2.

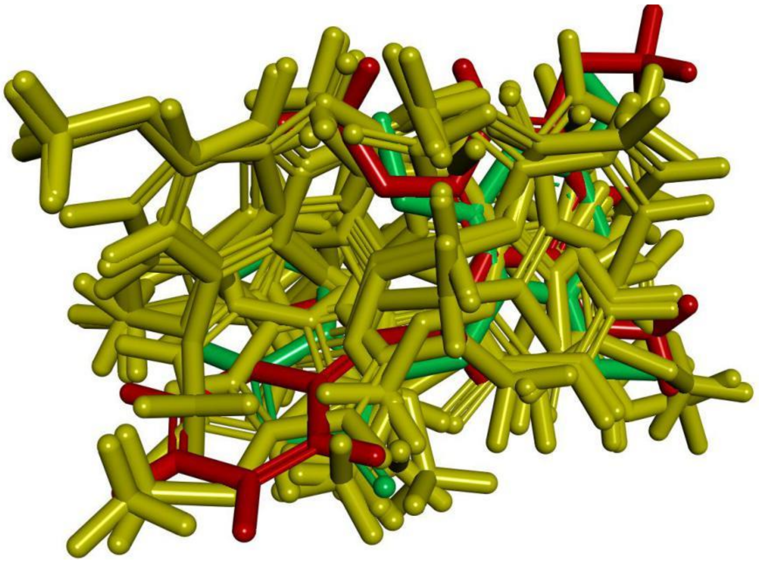

Figure S1. The superimposition of the original and the redocking conformations of IC261. The original conformation is shown in green; the optimal conformation is shown in red; and other conformations are shown in yellow.

```
Heavy Atom RMSD to Original-IC261 12

Name                          Reference                     RMSD (A)
261_2.1_refmac2 1             Original-IC261 12               2.3645
261_2.1_refmac2 2             Original-IC261 12               3.7589
261_2.1_refmac2 3             Original-IC261 12               5.6993
261_2.1_refmac2 4             Original-IC261 12               6.1975
261_2.1_refmac2 5             Original-IC261 12               5.7047
261_2.1_refmac2 6             Original-IC261 12               5.7432
261_2.1_refmac2 7             Original-IC261 12               6.4057
261_2.1_refmac2 8             Original-IC261 12               5.7875
261_2.1_refmac2 9             Original-IC261 12               5.9292
261_2.1_refmac2 10            Original-IC261 12               4.0373
Original-IC261 12             Original-IC261 12               0.0000
The following molecule(s) failed due to the number of atoms
not matching the reference ligand

261_2.1_refmac2               1
```

Figure S2. The root mean square variance (RMSD) between the docking conformation and the original conformation.
